# Phoenixin-20 ameliorates Sevoflurane inhalation-induced post-operative cognitive dysfunction in rats via activation of the PKA/CREB signaling

**DOI:** 10.18632/aging.205177

**Published:** 2023-12-04

**Authors:** Jing Zhang, Zhao Wang, Kun Cong, Jun Qi, Lining Sun

**Affiliations:** 1 Department of Anesthesiology, The Affiliated Yantai Yuhuangding Hospital of Qingdao University, Yantai 264000, Shandong, China

**Keywords:** phoenixin-20, Sevoflurane, post-operative cognitive dysfunction, PKA/CREB signaling

## Abstract

Post-operative cognitive dysfunction (POCD) is a common complication after surgery due to the usage of anesthetics, such as Sevoflurane, which severely impacts the life quality of patients. Currently, the pathogenesis of Sevoflurane-induced POCD has not been fully elucidated but is reportedly involved with oxidative stress (OS) injury and aggravated inflammation. Phoenixin-20 (PNX-20) is a PNX peptide consisting of 20 amino acids with promising inhibitory effects on OS and inflammation. Herein, we proposed to explore the potential protective function of PNX-20 on Sevoflurane inhalation-induced POCD in rats. Sprague-Dawley (SD) rats were treated with 100 ng/g PNX-20 for 7 days with or without pre-inhalation with 2.2% Sevoflurane. Markedly increased escape latency and decreased time in the target quadrant in the Morris water maze (MWM) test, and aggravated pathological changes and apoptosis in the hippocampus tissue were observed in Sevoflurane-treated rats, which were markedly attenuated by PNX-20. Furthermore, the aggravated inflammation and OS in the hippocampus observed in Sevoflurane-treated rats were notably abolished by PNX-20. Moreover, the brain-derived neurotrophic factor (BDNF), protein kinase A (PKA), and phospho-cAMP response element binding protein/cAMP response element binding protein (p-CREB/CREB) levels were markedly decreased in Sevoflurane-treated rats, which were memorably increased by PNX-20. Our results indicated that PNX-20 ameliorated Sevoflurane inhalation-induced POCD in rats via the activation of PKA/CREB signaling, which might supply a new treatment approach for POCD.

## INTRODUCTION

PCOD is a common complication after surgery and mainly manifests as impaired cognitive ability, including attention, memory, information processing, and executive function. POCD seriously threatens the quality of life of patients, especially the elderly [1]. With the popularity of anesthesia/surgery, POCD has been paid high attention worldwide in recent years. It has been shown that, among patients with apparently good cognition before undergoing anesthesia and noncardiac surgery, about 12% develop symptoms of cognitive dysfunction after surgery [2]. In recent years, researchers have discovered that neuroinflammation, autophagy disorders, abnormal sleep-wake cycle, and intestinal microbial disorders may be correlated to the occurrence of POCD in the elderly, and a certain relationship is reported between the development of POCD and the usage of anesthesia [3, 4]. Sevoflurane is one of the most commonly used volatile anesthetics due to its advantages of fast inhalation, fast induction, and good controllability [5]. Although anesthesia greatly reduces the suffering of patients undergoing surgery, recent studies have shown that exposure to inhaled anesthetics induces neuropathological changes, such as neuronal apoptosis and increased Aβ protein levels [6, 7]. General anesthesia with volatile anesthetics alone is reported to produce abnormal social behaviors and POCD similar to autism spectrum disorder, and the neurotoxic effects of Sevoflurane may be mediated through neuroinflammation, neurotransmitter imbalance or decreased concentration of brain-derived neurotrophic factor (BDNF) [8]. Currently, the pathogenesis of Sevoflurane-induced POCD has not been fully elucidated, and it is believed to be related to OS injury, aggravated inflammatory response, hippocampal neuron apoptosis, and neurotransmitter abnormalities, among which OS injury and inflammation are important mechanisms causing cognitive dysfunction [9]. Stress responses and excessive oxygen free radicals are produced by the inhalation of Sevoflurane, resulting in OS injury. LIU et al. [10] claimed that Sevoflurane enhanced OS injury and promoted neuronal apoptosis in the hippocampus of young rats. Several studies report that the pro-inflammatory cytokines IL-1β, IL-6, and TNF-α were statistically significantly increased in the brains of rats exposed to Sevoflurane [11, 12]. Sevoflurane was recently described to induce neuroinflammation and lead to POCD through increasing reactive oxygen species (ROS) and activating the Nod-like receptor protein 3 (NLRP3) inflammasome/NF-κB signaling pathway [13]. Therefore, exploring effective and safe drugs or methods to inhibit OS injury and inflammatory response will be novel directions for treating Sevoflurane-induced POCD in elderly patients.

Phoenixin (PNX), also known as SMIM20, is an endocrine peptide originally identified in the hypothalamus in 2013 and is involved in reproductive regulation. PNX enhances gonadotropin-releasing hormone, which in turn increases luteinizing hormone secretion and promotes the expression of Gonadotropin-releasing hormone receptor (GnRH-R) [14]. PNX and its receptor GPR173 are widely distributed in the hypothalamus and other tissues [15]. It is recently reported that the microinjection of PNX into the lateral ventricle significantly promotes food intake in rats [16], microinjection of PNX into the anterior hypothalamus (ANA) inhibits anxiety [17], and microinjection of PNX into the lateral ventricle or hippocampus alleviates learning and memory deficits in rodents [18]. PNX-20 is an PNX peptide with 20 amino acids and is recently reported to possess promising inhibitory effects on LPS-induced inflammatory response [19, 20]. Furthermore, Wang et al. reported that administration of PNX-20 possessed a neuroprotective effect by ameliorating brain infarction in an ischemic stroke model [21]. Herein, our study proposes to check the potential protective function of PNX-20 on Sevoflurane inhalation-induced POCD in rats.

## RESULTS

### GPR173 was upregulated in the hippocampus tissue of Sevoflurane-treated rats

**Figure 1:**
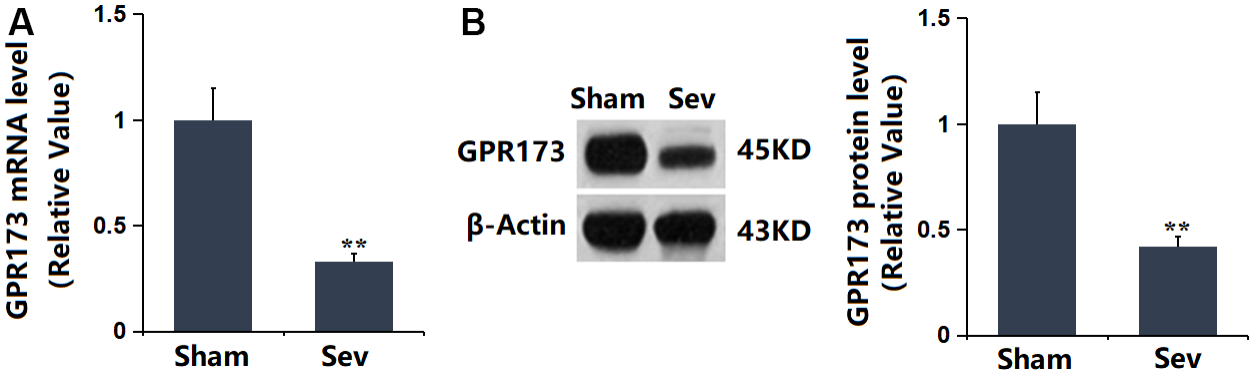
**The expression of GPR173 was repressed in the hippocampus tissue of Sevoflurane (Sev)-treated rats.** (**A**) mRNA of GPR173 was measured by real-time PCR; (**B**) Protein of GPR173 as measured by western blots (n=6, *, **, *P<*0.05 or 0.01 vs. sham group).

To explore the potential role of GPR173 in Sevoflurane-induced POCD, the GPR173 level was checked in the hippocampus tissue. It was found that GPR173 was markedly downregulated in the hippocampus tissue of Sevoflurane-treated rats (Figure 1A, 1B), implying that targeting GPR173 might be effective in treating Sevoflurane-induced POCD.

### PNX-20 attenuated Sevoflurane-induced learning and memory impairments in rats

**Figure 2:**
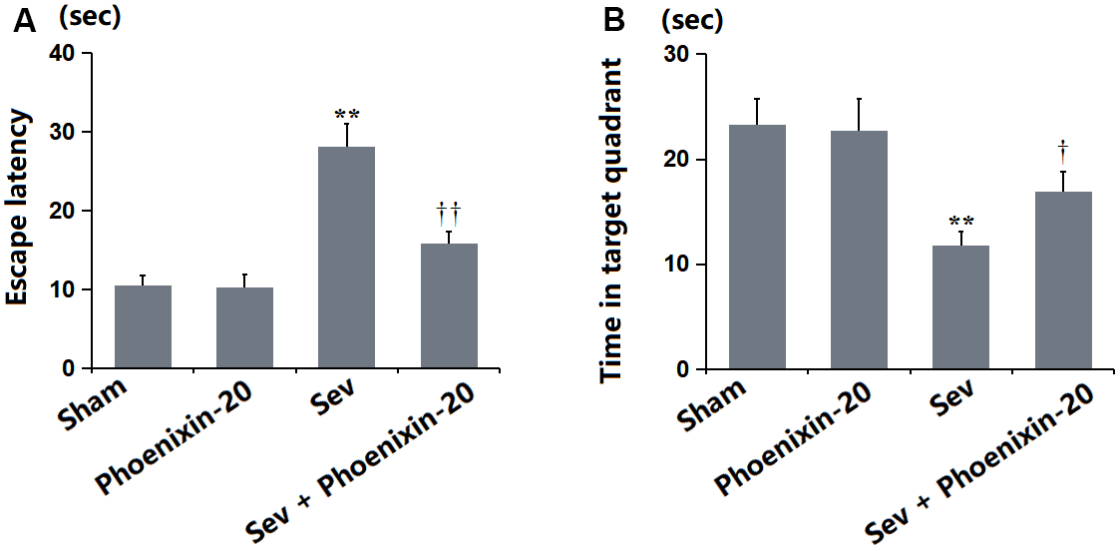
**Phoenixin-20 attenuated Sev-induced learning and memory impairments in rats.** (**A**) Escape latency (sec). (**B**) Time in target quadrant (sec) (n=6, *, **, *P<0.05* or 0.01 vs. sham group; †, ††, *P<*0.05 or 0.01, vs. Sev group).

SD rats were treated with 100 ng/g PNX-20 for 7 days with or without pre-inhalation with 2.2% Sevoflurane, followed by testing the learning and memory function using the MWM experiment. The escape latency was maintained around 10.5 s in the Sham and PNX-20 groups but was largely increased to 28.1 s in the Sev group, which was markedly reduced to 15.8 s by PNX-20 (Figure 2A). Furthermore, the time in the target quadrant in the Sham, PNX-20, Sev, Sev+ PNX-20 groups was 23.3, 22.7, 11.8, and 16.9 s, respectively (Figure 2B). The learning and memory functions impaired in Sevoflurane-treated rats were alleviated by PNX-20.

### PNX-20 alleviated the neuronal damage in the hippocampus tissue of Sevoflurane-treated rats

**Figure 3:**
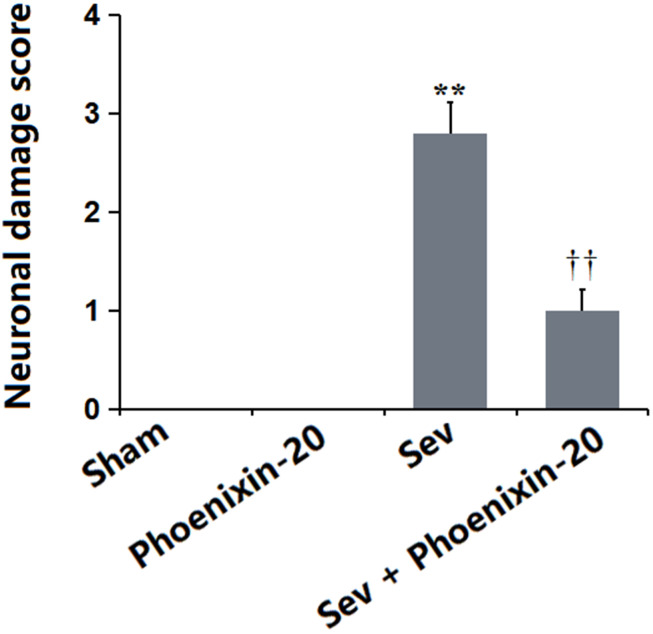
**Phoenixin-20 alleviated the neurodamage in the hippocampus tissue of Sev-treated rats.** Neuronal damage score (n=6, *, **, *P<0.05* or 0.01 vs. sham group; †, ††, *P<*0.05 or 0.01, vs. Sev group).

The neuronal damage score in the Sham and PNX-20 groups was kept at 0 but was notably increased to 2.8 in the Sev group, which markedly declined to 1.0 in the Sev+ PNX-20 group (Figure 3). A protective property of PNX-20 against hippocampus damage in Sevoflurane-treated rats was observed.

### PNX-20 alleviated the apoptosis in the hippocampus tissue of Sevoflurane-treated rats

**Figure 4:**
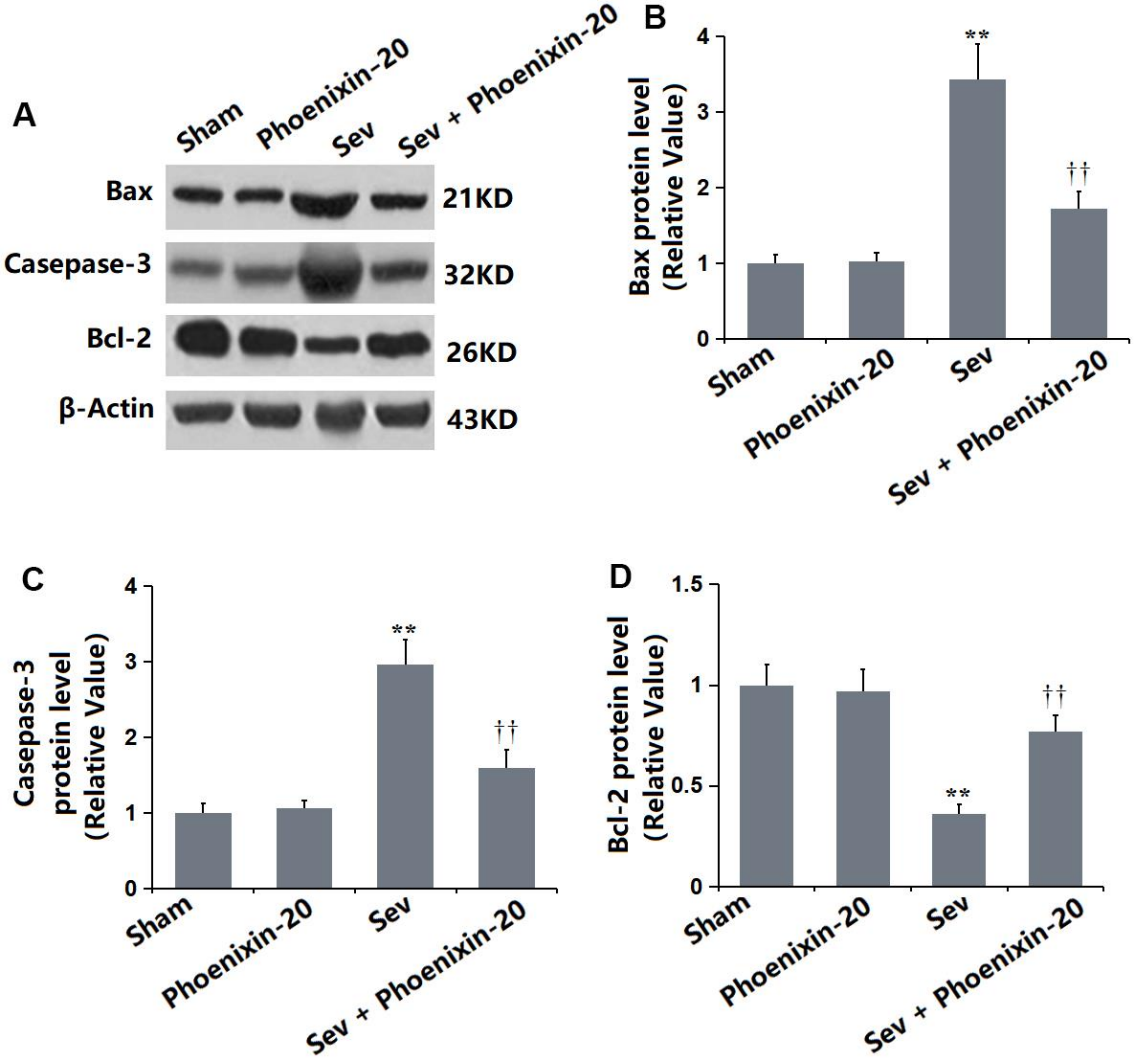
**Phoenixin-20 alleviated the apoptosis in the hippocampus tissue of Sev-treated rats.** (**A**) Protein level was determined using western blots. (**B**) Analysis of (**A**) Bax, (**C**) Casepase-3 and (**D**) Bcl-2 (n=6, *, **, *P<0.05* or 0.01 vs. sham group; †, ††, *P<*0.05 or 0.01, vs. Sev group).

To check the apoptotic status in the hippocampus tissue, levels of apoptosis-related proteins were detected. As illustrated in Figure 4A–4C, the Bax, Casepase-3, and Bcl-2 levels were kept unchanged in the Sham and PNX-20 groups. Bax and Casepase-3 were signally upregulated, while Bcl-2 was markedly downregulated in the Sev group, which were observably reversed by 100 ng/g PNX-20.

### PNX-20 repressed the release of inflammatory cytokines in the hippocampus tissue of Sevoflurane-treated rats

**Figure 5:**
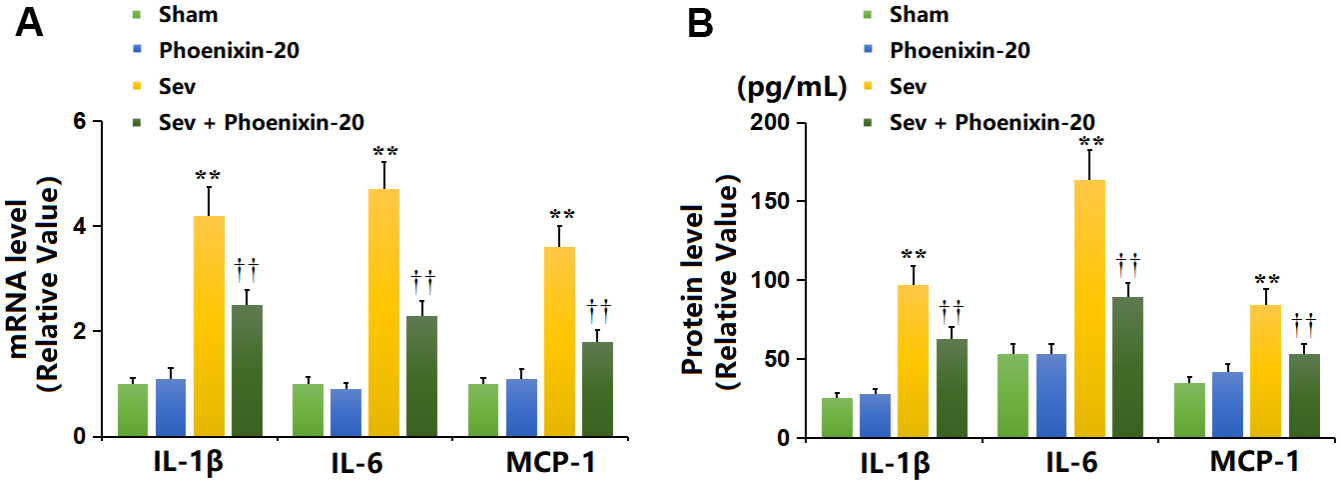
**Phoenixin-20 repressed the release of inflammatory cytokines in the hippocampus tissue of Sev-treated rats.** (**A**) mRNA level of IL-1β, IL-6, and MCP-1. (**B**) Protein level of IL-1β (pg/mL), IL-6 (pg/mL), and MCP-1 (pg/mL) (n=6, *, **, *P<0.05* or 0.01 vs. sham group; †, ††, *P<*0.05 or 0.01, vs. Sev group).

It was found that the mRNA levels of IL-1β, IL-6, and MCP-1 in the hippocampus tissue were maintained unchanged in the PNX-20 group, largely elevated in the Sev group, then memorably reduced in the Sev+ PNX-20 group (Figure 5A). The IL-1β content (Figure 5B) in the Sham, PNX-20, Sev, Sev+ PNX-20 groups was 25.5, 28.1, 97.2, and 62.8 pg/mL, respectively. The IL-6 level was minorly changed from 53.2 to 52.9 pg/mL in the PNX-20 group but was largely increased to 163.8 pg/mL in the Sev group, which was markedly repressed to 89.3 pg/mL in the Sev+ PNX-20 group. Moreover, the release of MCP-1 in the Sham, PNX-20, Sev, Sev+ PNX-20 groups was 34.7, 41.9, 84.5, and 52.8 pg/mL, respectively. A marked inhibitory effect of PNX-20 on inflammation in the hippocampus tissue of Sevoflurane-treated rats was observed.

### PNX-20 mitigated the OS in the hippocampus tissue of Sevoflurane-treated rats

**Figure 6:**
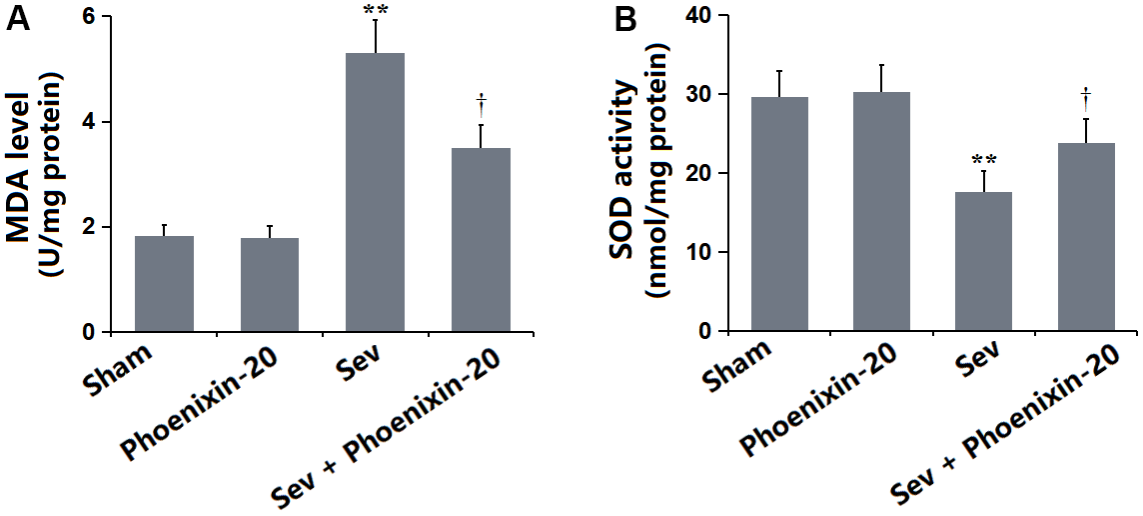
**Phoenixin-20 mitigated the oxidative stress in the hippocampus tissue of Sev-treated rats.** (**A**) MDA level (nmol/mg protein). (**B**) SOD activity (U/mg protein) (n=6, *, **, *P<0.05* or 0.01 vs. sham group; †, ††, *P<*0.05 or 0.01, vs. Sev group).

OS is a critical pathological mechanism involved in Sevoflurane-induced POCD [22]. The MDA level in the PNX-20 group was slightly changed from 1.82 to 1.78 nmol/mg protein but was markedly increased to 5.31 nmol/mg protein in the Sev group, which was notably reduced to 3.49 nmol/mg protein by 100 ng/g PNX-20 (Figure 6A). Furthermore, the SOD activity in the Sham, PNX-20, Sev, Sev+ PNX-20 groups was 29.7, 30.3, 17.6, and 23.8 U/mg protein, respectively (Figure 6B). A dramatically repressive effect of PNX-20 on OS in Sevoflurane-treated rats was observed.

### PNX-20 increased the level of BDNF in the hippocampus tissue of Sevoflurane-treated rats

**Figure 7:**
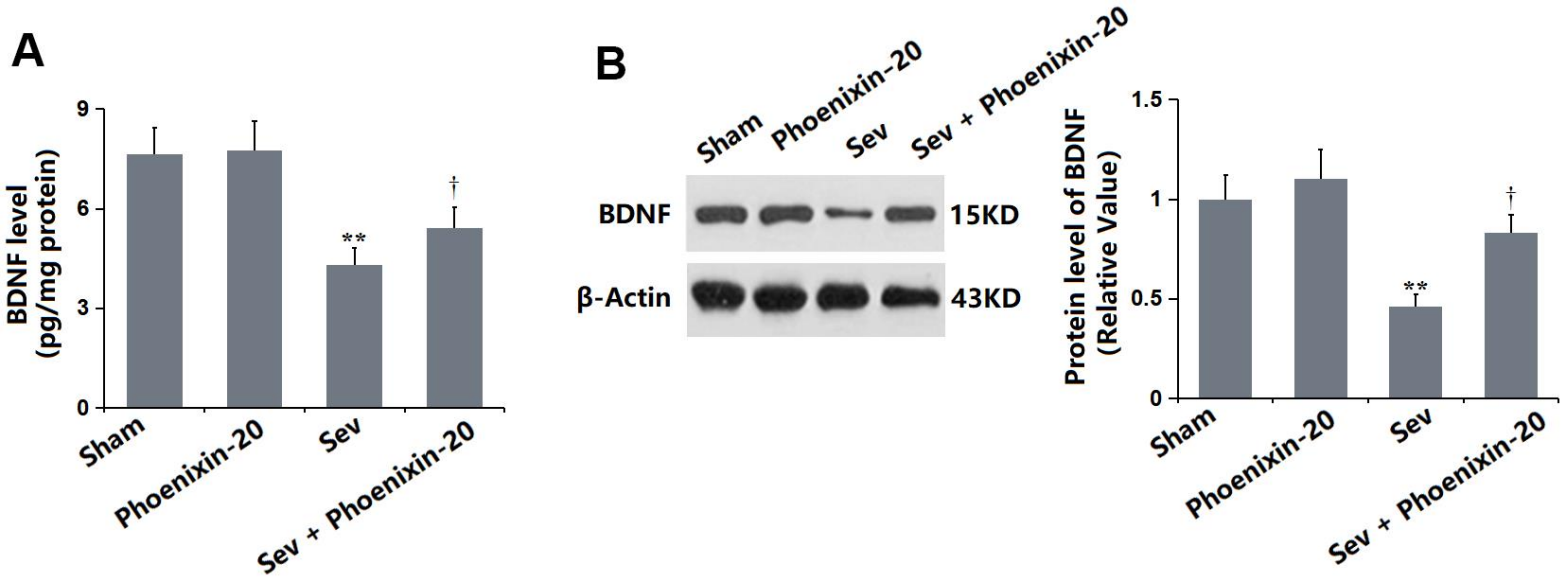
**Phoenixin-20 increased the level of BDNF in the hippocampus tissue of Sev-treated rats.** (**A**) BDNF level in the hippocampus tissue (pg/mg protein). (**B**) Protein level of BDNF was measured by Western blotting (n=6, *, **, *P<0.05* or 0.01 vs. sham group; †, ††, *P<*0.05 or 0.01, vs. Sev group).

BDNF is a critical biomarker for evaluating the learning and memory function [23]. The BDNF level in the PNX-20 group was minorly altered from 7.63 to 7.75 pg/mg protein but was prominently decreased to 4.29 pg/mg protein in the Sev group, which was notably promoted to 5.41 pg/mg protein by 100 ng/g PNX-20 (Figure 7A). The protein level of BDNF checked by Western blotting in each group was consistent with data obtained from the ELISA (Figure 7B).

### PNX-20 activated PKA/CREB signaling in the hippocampus tissue of Sevoflurane-treated rats

**Figure 8:**
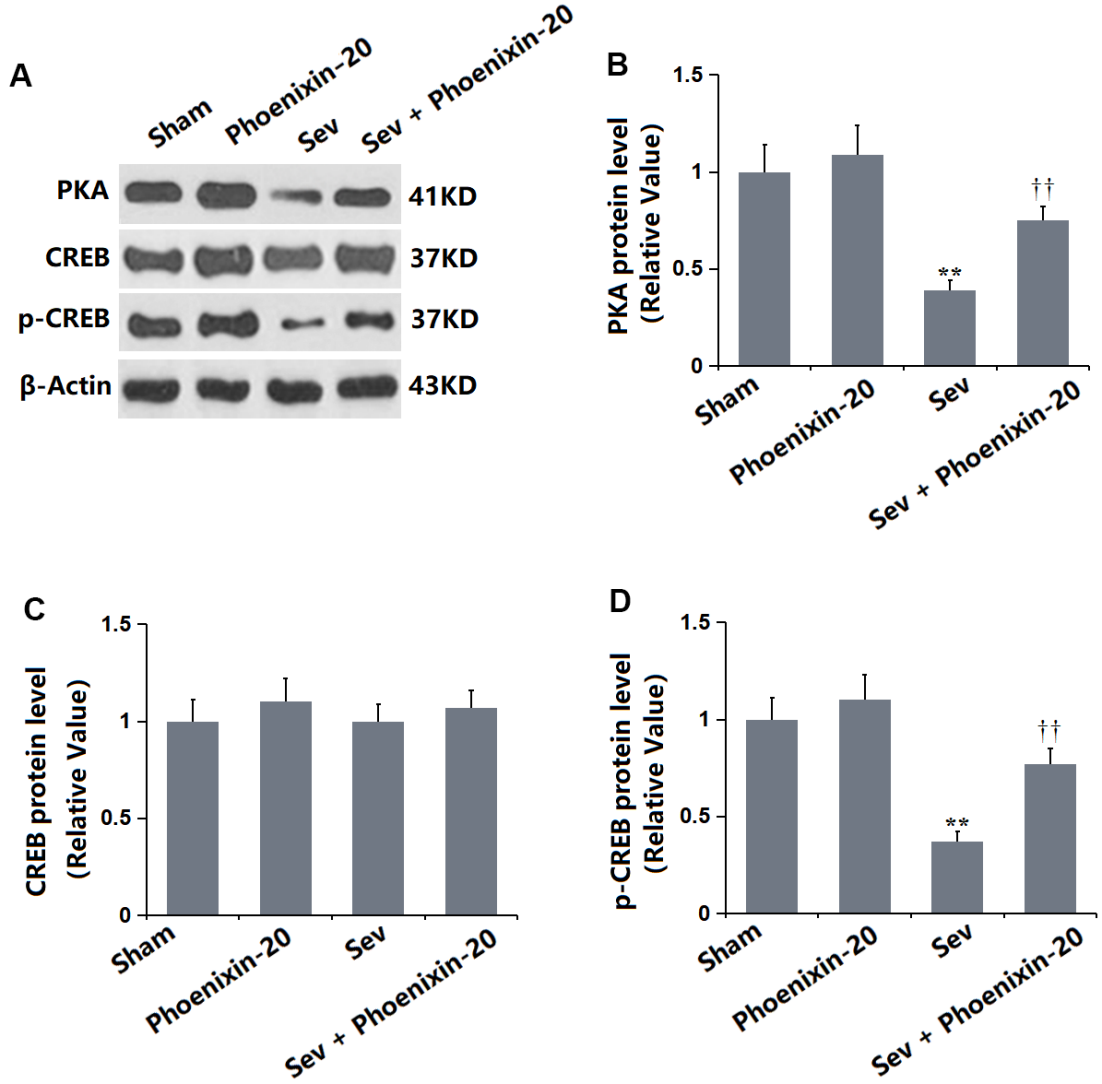
**Phoenixin-20 activated the PKA/CREB signaling in the hippocampus tissue of Sev-treated rats.** (**A**) Protein level was determined using western blots. Analysis of (**B**) PKA; (**C**) CREB; (**D**) Protein level of p-CREB (n=6, *, **, *P*<0.05 or 0.01 vs. sham group; †, ††, *P*<0.05 or 0.01, vs. Sev group).

PKA/CREB signaling is reported to regulate BDNF expression in the hippocampus [24]. It was found that the PKA (Figure 8A) and p-CREB/CREB levels (Figure 8B) were slightly altered in the PNX-20 group, but signally repressed in the Sev group, then markedly increased in the Sev+ PNX-20 group. PKA/CREB signaling in Sevoflurane-treated rats was activated by PNX-20.

## DISCUSSION

POCD refers to the persistent impairment of memory, abstract thinking, and orientation in patients undergoing anesthesia and surgery, accompanied by a decrease in social activities, such as changes in personality, social ability, cognitive ability, and postoperative skills [25]. POCD has become a major health problem in postoperative anesthesia and there is an urgent need to clarify the correlation between anesthetic exposure and the occurrence of postoperative cognitive impairment. The hippocampus is a part of the brain directly related to cognition, learning, and memory. The damage to hippocampal structure results in cognitive dysfunction, and the decline of learning and memory ability. Studies [26, 27] have shown that the activation of inflammation and OS in the hippocampus is directly correlated to the structural damage during cognitive impairment induced by Sevoflurane. In our study, consistent with data presented by Wei [28], impaired learning and memory function, and pathological changes and apoptosis in the hippocampus tissue were observed in Sevoflurane-treated rats, which were markedly attenuated by PNX-20, implying a protective function of PNX-20 against POCD in Sevoflurane-treated rats. Moreover, in line with the observation in mice reported by Gao [29], aggravated inflammation and OS in the hippocampus were observed in Sevoflurane-treated rats, which were sharply alleviated by PNX-20, suggesting that the protective property of PNX-20 might be correlated with the repressive effects on inflammation and OS.

cAMP is an important second messenger in cAMP/PKA/CREB signaling [30]. PKA is a key downstream target of cAMP, which regulates the survival and development of neurons to maintain synaptic plasticity and induce long-term memory formation, as well as mediate a variety of biochemical reactions in cells. Intracellular cAMP activates PKA and stimulates downstream factors to promote nerve regeneration [31]. PKA catalyzes the phosphorylation of CREB by the serine 133 subunit. CREB exists in various neurons of the brain and is a regulator in the nucleus of eukaryotic organisms. It plays a regulatory role in neurophysiological activities, such as neuronal development, regeneration, and synaptic plasticity, closely correlated to learning and memory ability [32]. Studies have shown that expression of the NMDA receptor is upregulated by the activation of the PKA-CREB pathway, and learning and memory are related to the promotion of postsynaptic long-term potentiation [33–35]. p-CREB is crucial for the formation of hippocampus-dependent long-term memory and is the intersection of multiple signal transduction pathways. By stimulating noradrenergic receptors and serotonin receptors, the c-AMP-PKA pathway is activated to increase the p-CREB level [36]. CREB is reported to regulate the downstream synthesis of BDNF, thereby promoting neuronal repair and inhibiting neuronal apoptosis [37]. BDNF is one of the downstream target genes of CREB and is the most important target for observing changes in hippocampal synaptic plasticity. It participates in neuronal survival and promotes synaptic transmission and occurrence in the central nervous system. BDNF is widely distributed and the upregulation of BDNF is essential for the survival of neurons and improving cognitive function [38]. In our research, the repaired learning and memory function in Sevoflurane-treated rats was accompanied by inactivated PKA/CREB signaling and a reduced BDNF level, which were signally reversed by PNX-20, implying the protective function of PNX-20 might be correlated with the activation of PKA/CREB/BDNF signaling. In upcoming work, the mechanism will be identified by co-administering Sevoflurane-treated rats with PNX-20 and an inhibitor of PKA/CREB/BDNF signaling.

Collectively, PNX-20 ameliorated Sevoflurane inhalation-induced POCD in rats via the activation of PKA/CREB signaling.

## MATERIALS AND METHODS

### Animals and grouping

Twenty-four SD rats were obtained from Charles River (Beijing, China) and were assigned into 4 groups: Sham (inhaled with 40% oxygen), PNX-20 (injected with 100 ng/g PNX-20 for 7 days), Sev (inhaled with 2.2% Sevoflurane), Sev + Phoenixin-20 (inhaled with 2.2% Sevoflurane and injected with 100 ng/g PNX-20 for 7 days) [21, 22].

### Reverse-transcription polymerase chain reaction (RT-PCR) assay

To harvest hippocampus tissues, the rats were placed in clean, transparent, and sealed containers connected to a CO_2_ Tanker, until they could not move. Then, the rats were extracted and sacrificed immediately by cervical disassociation, one by one. Hippocampus tissues were lysed using the Trizol reagent for the extraction of total RNAs, followed by transforming isolated RNAs into cDNAs with a commercial kit (MedChemExpress, Monmouth Junction, NJ, USA). After conducting the PCR reaction using the 2×RealStar Fast SYBR qPCR Mix kit (GenStar, China), the 2^−ΔΔCt^ method was utilized for the determination of gene expressions. The primer sequences are listed in Table 1.

**Table 1:** Primer sequences.

	**Forward (5'-3')**	**Reverse (5'-3')**
GPR173	TCTGGTCACCCTACATCGTG	CAGTAGGGTTCTCTGGGAGC
IL-1β	GCAACTGTTCCTGAACTCAACT	ATCTTTTGGGGTCCGTCAACT
IL-6	GACAAAGCCAGAGTCCTTCAGAGAGATACAG	TTGGATGGTCTTGGTCCTTAGCCAC
MCP-1	CAAACTGAAGCTCGCACTCTCGCC	CTTGGGTrGTGGAGTGAGTGTTCA
GAPDH	GCACCGTCAAGGCTGAGAAC	TGGTGAAGACGCCAGTGGA

### The Morris water maze (MWM) test

After 7 days of treatment, a hidden platform placed in a pool (100 cm×40 cm) was used for the swim training, with sessions recorded. The motion detection software was utilized for the analysis of data. During the first four days, the spatial acquisition staining and testing sessions were performed on the rats 4 times. Then, the animals were put in fixed positions in the pool to seek the hidden platform within 1 min. The escape latency was defined as the time spent to locate the platform, which was recorded by the motion detection software. Subsequently, on the 5^th^ day, the platform was cleared and the probe testing was performed. The time spent (in one minute) in the quadrant where the platform was placed was recorded as the time in the target quadrant.

### Western blotting assay

Hippocampal tissues were added with RIPA lysate buffer (Cat#: R0278, Sigma-Aldrich, St. Louis, MO, USA), and homogenized on ice using a glass homogenizer, followed by extracting total proteins and measuring the protein concentration using the BCA method. Each well in the 12% SDS PAGE was loaded with 20 μg of protein for separation and then the proteins in the gel were transferred onto the PVDF membrane. After transfer, membranes were blocked with 5% skim milk and primary antibodies against Bax (Cat#41162, 1:2000, CST, Boston, MA, USA), Casepase-3 (Ca##14220, 1:1000, CST, USA), Bcl-2 (Cat#4223, 1:2000, CST, USA), BDNF (Cat#16696, 1:800, CST, USA), PKA (Cat#Ziker-0520R, 1:1000, ZIKER Bio Tech, Shenzhen, China), CREB (Cat#9197, 1:800, CST, Boston, USA), p-CREB (Cat#9198, 1:1000, CST, USA), and β-actin (Cat#3700, 1:2000, CST, USA) were applied to be incubated with the membrane. Subsequently, membranes were cultured with the secondary antibody (Cat#7074 or Cat#7076, 1:5000, CST, USA) and then the ECL luminescence solution (Cat#12630, CST, USA) was introduced for exposure. The image J system was used to analyze the gray value of protein bands.

### Enzyme-linked immunosorbent (ELISA) assay

The commercial kit (Cat#EH4352, eBioScience, San Diego, CA, USA; Cat#ERA31RBX10, Invitrogen, Carlsbad, CA, USA; Cat#EP16RB, Invitrogen, USA; Cat#ERBDNF, Invitrogen, USA) was applied for the detection of the concentrations of interleukin-1β (IL-1β), interleukin-6 (IL-6), monocyte chemoattractant protein-1 (MCP-1), and BDNF in the hippocampal tissue, with kit instructions strictly followed. The hippocampal tissue was homogenized and centrifuged to obtain the supernatants, which were added to the 96-well plate, followed by incubation at 37° C for 1 hour and washing 5 times. Following adding enzyme-labeled reagents, samples were cultured at 37° C for half an hour. Then, the chromogenic solutions A and B were introduced and cultured at 37° C for 10 min. Following adding the termination solution, the OD value was read to calculate the concentrations.

### The measurement of malondialdehyde (MDA) level and superoxide dismutase (SOD) activity

The MDA level in the hippocampal tissue was measured utilizing the TBA method with the commercial kit (Qingdao Jisskang Biotechnology, China), while the nitroblue tetrazolium (NBT) method was applied for the detection of SOD activity in the hippocampal tissue using the commercial kit (Beyotime, China). All steps were strictly followed according to the kit instructions.

### Statistical analysis

Experiments were repeated for at least 3 times. Achieved data were expressed as mean±standard deviation (SD) and were analyzed with the GraphPad software. The comparison was performed using the one-way analysis of variance (ANOVA) method with Tukey’s test. P<0.05 was regarded as a significant difference.

### Data availability

The data is available on request from the corresponding authors.
